# Heavy Metal Pollution and Potential Ecological Risk Assessment in a Typical Mariculture Area in Western Guangdong

**DOI:** 10.3390/ijerph182111245

**Published:** 2021-10-26

**Authors:** Ning He, Lanzhou Liu, Ren Wei, Kaifeng Sun

**Affiliations:** 1 School of Life Science and Resources Environment, Yichun University, Yichun 336000, China; hening2010@jxycu.edu.cn (N.H.); liulanzhou2020@163.com (L.L.); weir2020@163.com (R.W.); 2 School of Ecology and Environment, Inner Mongolia University, Huhhot 010021, China; 3 South China Institute of Environmental Sciences, Ministry of Ecology and Environment, Guangzhou 510655, China

**Keywords:** heavy metals, mariculture environment, potential ecological risk assessment, offshore pollution evaluation

## Abstract

The distribution characteristics, environmental contamination states, and potential ecological risks of chromium (Cr), copper (Cu), arsenic (As), lead (Pb), and cadmium (Cd) in seawater, sediment and breeding feed were studied in a typical mariculture area in western Guangdong of China. Pearson correlation analysis was used to determine metal homology, and the single-factor index, potential ecological risk index, hazard quotient (HQ), and joint probability curve (JPC) were used to evaluate pollution states and ecological risk of metals. Four main statements can be concluded from the results: (1) Pb and Cu showed a similar distribution pattern in the seawater and sediment and their contents in the breeding wastewater exceeded the standard limits in several stations. (2) Cr, Cu, and As have similar sources in the feeds, which may be an important source of metals in water. (3) The risk assessment revealed that the sediment from the studied areas was at a low ecological risk of heavy metal, whereas, water in the pond and outfall was slightly polluted by Pb, and water in the cages and outfall were slightly polluted by Cu. (4) Both the hazard quotient (HQ) and joint probability curve showed the overall risk probabilities (ORPs) in the waters ranked as Cu > Cr > Pb > Cd > As. Although Pb and Cd had HQ values greater than 1, their ORPs were acceptable. This study highlights that multiple evaluation models are more reliable than the single ecological risk assessment for evaluating heavy metal pollution risks in the mariculture area.

## 1. Introduction

Coastal ecosystems provide considerable services and support for the development of human society, including cultural service, provisioning service, regulating service, and supporting service. The support offered by provisioning services has continuously increased in recent years [1]. China’s total catch volume is approximately 15 million tons (ocean fishing yields up to 13 million tons), which accounted for 15% of the total global catch volume in 2018 [2]. However, due to terrestrial urbanization and the rapid growth of coastal port development and trade, energy production, and aquaculture, large amounts of industrial and agricultural wastewater and urban domestic sewage are discharged into the offshore environment posing serious threats to the marine environmental quality and local ecological security [3,4]. Heavy metals enter the ocean via several major sources, most notably riverine influx, atmospheric deposition, and anthropogenic activities [5,6]. Organisms are exposed to heavy metals mainly through the food chains and skin contact resulting in great risks to human health [7,8]. Heavy metals have continued to attract considerable attention worldwide due to their bioaccumulation, difficult degradation, and the high latency of their effects [9,10,11]. Cd in submarine sediment poses a moderate potential ecological risk to coral islands and reefs in the Persian Gulf [12]. Sediments in the northern Beibu Gulf were characterized by slight to moderate pollution by Hg, Cu, Cr, Cd, As, and Zn [13]. In the mariculture area of Zhelin Bay, the Cr, Cu, and Cd levels in sediment all meet the marine sediment class I standards, but the Pb levels fall within the class II of the standard, and the heavy metal ecological risk was between mild and moderate [14]. At all sites in Xiangshan Bay, As in the sediment exceeded the Effects Range-Low (ERL, NOAA) safe levels and dissolved Pb and Hg were the major pollutants in the seawater [15]. In western Laizhou Bay, the hazard quotients (HQs) for Cr, Cu, and Zn were greater than 1, and the overall risk probabilities (ORPs) of their adverse effects were higher than 0.05, suggesting ecological risk. Specifically, the ORP of As was lower than 0.05, namely its ecological risk was acceptable [16]. In northern Liaodong Bay, the ecological risks posed by heavy metals in seawater were relatively low with only Cu and Pb levels exceeding the criteria maximum concentration set by National Oceanic and Atmospheric Administration (NOAA) (2004) in spring, and Cu also exceeded the criteria maximum concentration in winter [17]. The concentrations of As and Hg in the Persian Gulf were found exceeding the limits considered hazardous for aquatic life, and Hg posing the greatest potential ecological risk in the sediment [18].

Western Guangdong is one of the main mariculture areas in Guangdong Province, China. Studies on heavy metals related to this mariculture area in this area have been focused on detecting marine organisms [19,20], while there has been a lack of research on heavy metals in the breeding environment, impeding the study of its harmful effects of heavy metals on aquatic life based on environmental factors. Moreover, the pond breeding and cage breeding methods were adopted in western Guangdong, making the spatial position of the breeding subjects during culture relatively fixed. Once the culturing environment is polluted, the quality of aquatic products tends to notably respond to changes in the aquaculture environment, which puts the regional biodiversity at risk. This paper analyzed the distribution characteristics, correlations, environmental pollution levels, and potential ecological risks of heavy metals in seawater and sediment samples collected from typical farming ponds and Jida port breeding cages in Dianbai District, Maoming City, Guangdong Province. The goal is to determine the pollution status and ecological risks in the breeding area of western Guangdong to provide scientific guidance for the sustainable development of mariculture and data support for implementing mariculture production standards.

## 2. Materials and Methods

### 2.1. Study Area

**Figure 1:**
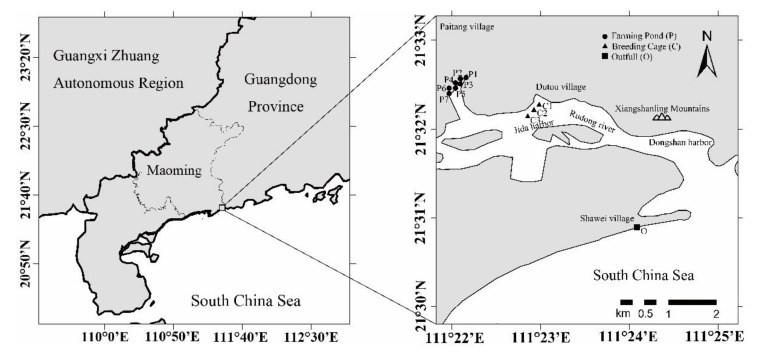
Overview of the study area.

The study area is Dianbai District, Maoming City, located in the eastern part of western Guangdong. Dianbai District has a 2128 km^2^ land area and a 1132 km^2^ sea area with an average depth of 20 m (including a 480 km^2^ sea area with a depth of 10 m). The sea area of Dianbai District extends to the coastline in the north and Fangji Bay in the south (111°1′51″ E~111°20′18″ E, 21°20′18″ N~21°25′12″ N). The intertidal mudflat, which is approximately 10,000 hectares, is a typical mariculture area in western Guangdong (Figure 1).

### 2.2. Sample Collection

In July 2018, the project team conducted a field survey and field sampling in the coastal areas of Dianbai District, with sampling stations distributed in 11 blocks, which covered seven artificial farming ponds (P1, P2, P3, P4, P5, P6, and P7), three breeding cages (C1, C2, C3) in Jida Harbor and the total outfall (O) in Shawei Beach (Figure 1). Three representative stations were chosen in each block, and three parallel samples were collected from each station and then mixed well in a clean polyvinyl bucket. Following the Specification for Offshore Environmental Monitoring (HJ 442-2008), the seawater samples were collected using a liquid sampler made of plexiglass. The collected water samples were filtered through a 0.45 µm acid-treated Millipore filter to remove impurities, and adjusting the pH to below 2 by adding hydrogen nitrate (1:1, GR) for subsequent laboratory analysis, then preserved in clean polyvinyl bottles at low temperature in the dark.

In the field, dissolved oxygen (DO) was measured with a portable dissolved oxygen meter, the pH was measured with a pH meter, and seawater temperature was measured with a special thermometer used for aquaculture (for evaluating DO). Sediment samples were collected from artificial farming ponds and breeding cages using a Peterson grab dredger (30 cm × 15 cm). The collected sediment samples were preserved in a cold room, and then transported to a freezer and stored at −20 °C in the laboratory. As fishing feeds were being fed to prawns and tilapias in studied breeding farms and breeding cages, sampling nine fishing feed samples from seven farmers who responsible for feeding in local mariculture area, and packaged feed samples in plastic Ziplock bags.

### 2.3. Sample Processing and Analysis

Numerous results of metal ecological risk in the coastal areas of China [3,4,5,9,13,14,15,16,17] showed that the contents of metal Cr, Cu, As, Cd, and Pb in seawater and sediment tend to exceed national standard limits, additionally, these five metals have adversely biological effects on the coastal ecosystems more serious than other metals. Thus, the levels of the Cr, Cu, As, Cd, and Pb metals in seawater and sediments were selected for analysis according to the Specification for Marine Monitoring Part 4, Seawater Analysis (GB 17378.4-2007), and Part 5, Sediment Analysis (GB 17378.5-2007). As exploring metal relations in feed samples is one of the aims of this research, the five metal levels in feed were measured according to the normative reference documents of the Nuisanceless Food Safe Limit in Matching Feed for Fishing (NY5072-2002). Detailed metal analysis procedures were performed according to related articles [4,15,17]. All the sediment samples were first oven-dried with an air circulating oven at 100 °C for 24 h, and then, gravel and large debris were removed with tweezers. Next, the sediment samples were ground in an agate mortar and pestle, and sieved to obtain fractions below 96 µm. Then, 0.1 g sieved sediment sample was placed into a digestion tank, and then, 1 mL of 65% hydrogen nitrate was added and incubated for 4 h. Ultimately, 4 mL of 30% hydrogen peroxide was decanted, homogenized, and digested for 120 min in a microwave digestion system with 800 W power, and then, the volume was held constant for testing. The pretreatment of the feed samples was similar to that of the sediment samples. First, all the feed samples were oven-dried with an air circulating oven at 100 °C for 24 h. Next, the feed samples were ground in an agate mortar and pestle and then sieved to obtain a fraction below 96 µm. Then, 0.1 g sieved feed sample was placed into a digestion tank, and then, 1 mL of 65% hydrogen nitrate was added and incubated for 4 h. Finally, 4 mL of 30% hydrogen peroxide was decanted, homogenized, digested for 120 min in a microwave digestion system with 800 W power, and then, the volume was held constant for testing. For the seawater samples, 65% hydrogen nitrate and filtered seawater were mixed in a 1:12 ratio, and a mixed water sample containing 5% hydrogen nitrate was prepared for testing. In this research, the metals in all the samples were analyzed by means of inductively coupled plasma-mass spectrometry (ICP-MS 7700, Agilent, Santa Clara, CA, USA). For quality control, each batch of samples included a field blank sample and a whole-process blank sample. The field blank samples were designed according to GB17378.3-2007 and the actual sampling target, which aims to correct for contamination introduced during sampling, storing and transporting. Further, the whole-process blank samples were designed according to GB17378.4-2007, GB17378.5-2007, the normative reference documents of NY5072-2002 which aims to investigate contamination introduced during processing and testing. For each field blank sample or whole-process blank sample that was tested twice, the difference of last test value and previous test value is the error form field sampling or laboratory testing. The actual sample testing results are more reliable when testing value minus the sum of field error and whole-process error. Additionally, parallel samples were analyzed with all samples to eliminate accidental errors. The percentage recoveries for all heavy metals in the samples were between 93% and 106%, and the precision error was below 7%.

### 2.4. Evaluation Method

#### 2.4.1. Single-Factor Index

Given the heavy metal concentration in environmental matrices (e.g., sediments, water) are able to effected by background value, previous studies used the single-factor index (SFI) to eliminate the disturbance of background value. The SFI was calculated according to the ratio of environmental exposure concentration and standard limit concentration [21] as follows in Equation (1):

(1) $$P_{i}=C_{i}/S_{i}$$

where P*_i_* is the SFI of metal *i*, C*_i_* is the measured concentration of metal *i*, and S*_i_* is the standard limit concentration of metal *i*. The evaluation of metal levels in seawater and sediment from mariculture areas was performed following the Nuisance-Free Food Environmental Conditions of Seawater Farm Stations (NY 5362-2010). The background level of Cu in the South China Sea (0.05 mg·L^−1^) was chosen as a limit because there is no limit for Cu in NY 5362-2010 [22]. In addition, a level of 0.1 mg·L^−1^ Cu was chosen for evaluating the outfall in Shawei Beach based on the Water Drainage Standard for Seawater Mariculture (SC/T 9103-2007). P*_i_* ≤ 1 indicates no pollution, 1 < P*_i_* ≤ 2 indicates slight pollution, 2 < P*_i_* ≤ 3 indicates moderate pollution, and P*_i_* > 3 indicates serious pollution.

#### 2.4.2. Potential Ecological Risk Index

The potential ecological risk index is used to comprehensively analyze the ecological effects and toxicological properties of heavy metals in sediment based on sedimentary theory, heavy metal properties, environmental behavior, toxicity, and biological sensitivity [23]. The potential ecological risk index of heavy metal is calculated according to Equations (2) and (3):

(2) $$E_{j}^{i}=T_{i}\cdot P_{i}$$

(3) $${\mathrm{RI}}_{j}=\sum E_{j}^{i}$$

where P*_i_* is the SFI of metal *i*, details in (1). $T_{i}$ is the risk coefficient of metal *i*, and the risk coefficients of Cd, As, Cu, Pb, and Cr are 30, 10, 5, 5, and 2, respectively [24]. $E_{j}^{i}$ is the potential ecological risk factor of metal *i* at station *j*, and RI*_j_* is the potential ecological risk index of five heavy metals at station *j*. The evaluation criteria of $E_{j}^{i}$ and RI*_j_* are shown in Table 1.

#### 2.4.3. Hazard Quotient (HQ)

The HQ is used to evaluate ecological risk on the basis of the ratio of environmental exposure values to toxicity values. The ecological risk posed by a metal is acceptable when HQ < 1, but unacceptable, indicating the need for further assessment or measures to reduce risk, when HQ > 1 [25,26]. The HQ of a heavy metal is determined with Equations (4) and (5):

(4) $$\mathrm{PNEC}={\mathrm{HC}}_{5}/\mathrm{SF}$$

(5) HQ=EEC/PNEC

where EC is the predicted noneffective concentration of metal. HC_5_ is the 5% quantile of the species sensitivity distribution curve (SSD) of a metal. The SSD is a cumulative probability distribution model to fit biotoxicity data in order to show biological sensitivity to a contaminant [27]. Safety factor (SF) is set to a conservative value of 5. HQ is the hazard quotient of a metal, and EEC is the environmental exposure value of a metal.

#### 2.4.4. Joint Probability Curve (JPC)

JPCs indicate the probabilities of damage to species in a studied habitat, taking the cumulative probability from biotoxicity data as an argument and the anti-accumulation of the environmental exposure data as a dependent variable. A curve far from two coordinate axes reflects high ecological risk. The ORP of predicted hazardous biological effects is described as the area bounded by the curve and two coordinate axes [25]. The JPC of heavy metal is determined with Equation (6):

(6) $$\mathrm{ORP}=\int_{0}^{1}\mathrm{EXP}\left(x\right)\mathrm{dx}$$

where x is the level of damage to species by a metal, namely, harmful effects are observed for 100x% of the species; EXP(x) is the probability of harmful effects occurring. The environmental bioprotection level is usually set at 95% in the United States, the Netherlands, etc., and ecological risk is acceptable when the ORP of a contaminant to biomes is less than 0.05 [16,28].

### 2.5. Statistical Analysis

The data were statistically analyzed with Student’s t-test, the Anderson–Darling test, one-way ANOVA, and Pearson correlation analysis using IBM SPSS Statistics, Version 22.0. The HC_5_ and ORPs were calculated, and JPC models were built using MATLAB R2014b. Diagrams were drawn using OriginPro 2015, and the sampling station map was prepared using ArcMap10.6.

## 3. Results and Discussion

### 3.1. Heavy Metal Contents in Feed, Seawater and Sediment in the Mariculture Area

The mean concentrations of Cr, Cu, As, Cd, and Pb were 0.065 μg∙mL^−1^ (0.061–0.070), 0.026 μg∙mL^−1^(0.014–0.039), 0.003 μg∙mL^−1^(0.001–0.006), 3.14 × 10^−5^ μg∙mL^−1^ (6.2 × 10^−5^–1.3 × 10^−4^), and 0.019 μg∙mL^−1^ (0.003–0.067), respectively, in the seawater from the farming ponds. The mean concentrations of Cr, Cu, As, and Pb were 0.068 μg∙mL^−1^ (0.067–0.0.69), 0.055 μg∙mL^−1^ (0.047–0.059), 0.002 μg∙mL^−1^ (0.0021–0.0027), and 0.019 μg∙mL^−1^ (0.011–0.024), respectively, but Cd was not detected in the seawater from the breeding cages (Jida Harbor). The mean concentrations of Cr, As, Cd, and Pb in breeding water met the requirements of water quality for mariculture at the Nuisance-free Food Environmental Conditions in Seawater Farm Stations (NY 5362-2010), and the mean concentration of Cu was below the background level of Cu in the South China Sea. The mean concentrations of Cr, Cu, As, Cd, and Pb were 0.073 μg∙mL^−1^ (0.070–0.074), 0.181 μg∙mL^−1^ (0.181–0.181), 0.010 μg∙mL^−1^ (0.09–0.010), 8.58 × 10^−5^ μg∙mL^−1^ (8.31 × 10^−5^–8.62 × 10^−5^), and 0.052 μg∙mL^−1^ (0.051–0.053), respectively, in the water of the outfall (Shawei Beach), where the Cu and Pb levels exceeded the limits of the Water Drainage Standard for Seawater Mariculture (SC/T 9103-2007). The mean concentrations of Cr, Cu, As, Cd, and Pb were 6.778 μg∙g^−1^ (2.201–10.359), 2.748 μg∙g^−1^ (0.429–5.324), 0.483 μg∙g^−1^ (0.046–0.868), 0.023 μg∙g^−1^ (1.8 × 10^–4^–0.108), and 5.509 μg∙g^−1^ (2.653–9.949) in the sediment in the farming ponds. The mean concentrations of Cr, Cu, As, Cd, and Pb were 6.943 μg∙g^−1^ (6.820–7.066), 1.051 μg∙g^−1^ (0.763–1.338), 0.174 μg∙g^−1^ (0.065–0.283), 0.177 μg∙g^−1^ (0.108–0.245), and 6.563 μg∙g^−1^ (6.199–9.274), respectively, in the sediment from the breeding cages, averaged across all stations, which were below the sediment quality limits for mariculture in NY 5362-2010. In the nine breeding feed samples, the mean concentrations of Cr, Cu, As, Cd, and Pb were 1.76 μg∙g^−1^ (1.14–2.87), 18.65 μg∙g^−1^ (8.09–29.73), 0.79 μg∙g^−1^ (0.002–1.49), 0.83 μg∙g^−1^ (0.06–1.50), and 0.26 μg∙g^−1^ (0.001–0.85), respectively, which all meet the requirements in the No Public Nuisance Food Safe Limit in Matching Feed for Fishing (NY5072-2002).

**Figure 2:**
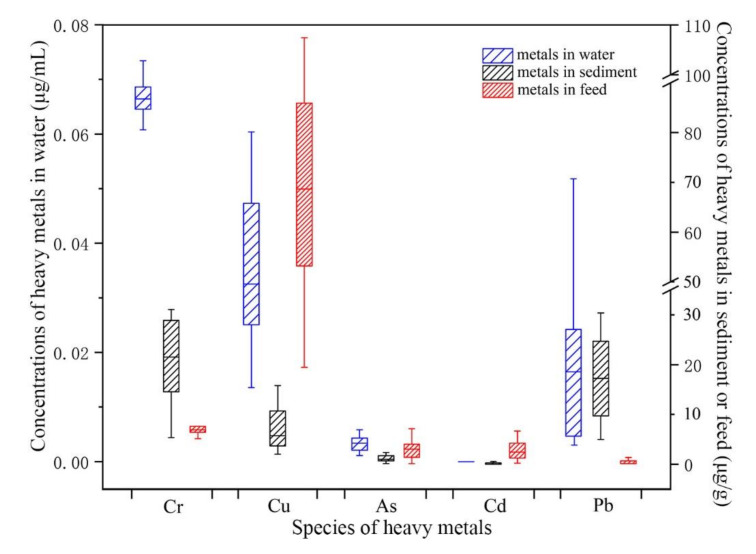
Distribution of the concentrations of five heavy metals in water, sediment and feed.

The distributions of the concentrations of the five metals in seawater and sediment were determined at thirty stations in the mariculture area and in nine breeding feed samples. The concentration ranges of Cu and Pb in seawater and in sediment were wide while those of As and Cd were narrow (Figure 2). The concentration range of Cr in water was narrower than that in sediment, perhaps because the physical and chemical conditions or breeding activities strongly impact the deposition of Cr in water. In addition, the content levels of Cr, As, Cd, and Pb in breeding feeds were below the standard limits, while there was no limit for Cu. Moreover, the concentration ranges of Cr, As, Cd, and Pb were narrow but the content of Cu was generally high and varied.

Compared with those in nonbreeding sea areas such as the Yalujiang Estuary [29], southern Yellow Sea [30], Jinzhou Bay [31], Tianjin Bay [32], Dingzi Bay [33], the Pearl River Estuary [34], the Changjiang Estuary [35], the southwest coast of the Bay of Bengal [36], Malaga Bay [37], and Laoshan Bay [38], the contents of Cu, Cr, and Pb in the seawater of the breeding area of Dianbai district were higher, but the contents of Cd and As were slightly lower or generally similar; these results indicate that mariculture affects Cu, Cr, and Pb levels more than As and Cd levels. The pH index and DO index were also used to evaluate water quality in the breeding areas of Dianbai District [39]. The results indicated that pH was slightly affected in all farming ponds and all clean in the breeding cages. Perhaps the relatively closed breeding space and high breeding density in farming ponds are conducive to the production and accumulation of some acidic materials, such as H_2_CO_3_ or H_2_S, leading to a low pH. However, the method of cage breeding in Jida Harbor is a semi-open breeding method, and the pH was similar to the background level in the marine environment and was less affected by the breeding process. The DO index evaluation showed that the DO level was not affected in all the breeding areas, but the mean DO concentration in the farming ponds was significantly higher than that in the cage breeding areas (*p* < 0.05); these results indicated that mechanical air exposure in ponds effectively increases the DO in water. There were extremely significant differences in the content of Cu in the farming ponds and breeding cages (*p* < 0.01), which may be a result of the seawater with low pH in the breeding cages in Jida Harbor, as heavy metals are more soluble in acidic water [40].

### 3.2. Correlation Analysis of Metals in Water, Sediment and Feed

**Figure 3:**
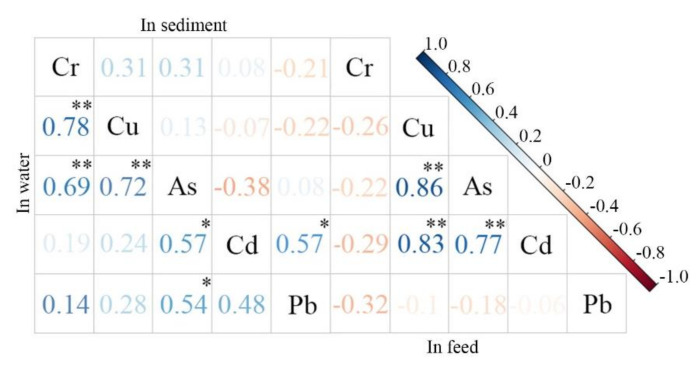
Correlations of metals in water, sediment and feed; Note: * represents a significant correlation, and ** represents an extremely significant correlation.

Pearson correlation analysis was used to analyze the correlations among the five metals in water, sediment, and feed in the maricultural area. The results showed correlations between various heavy metal concentrations in different matrices. In breeding water, the correlations of Cr-Cu (r = 0.78, *p* < 0.01), Cr-As (r = 0.69, *p* < 0.01), and Cu-As (r = 0.72, *p* < 0.01) were extremely significant and positive, and those of As-Cd (r = 0.57, *p* < 0.05) and As-Pb (r = 0.54, *p* < 0.05) were significant and positive (Figure 3). The sources of Cr, Cu, and As in water are probably same, and the pairs As-Cd and As-Pb may also have similar sources in water. In addition, all five heavy metals had a positive reciprocal association in water. Only Pb and Cd (r = 0.57, *p* < 0.05) were significantly positively correlated in sediment, and these metals may have similar chemical deposition properties. Moreover, the correlations of Cd-Cu (r = 0.83, *p* < 0.01), Cd-As (r = 0.77, *p* < 0.01), and Cu-As (r = 0.86, *p* < 0.01) in feeds were extremely significant and positive (Figure 3). The source of Cd, Cu, and As in feeds was the same, most likely dosed feed additives, which probably strengthened the correlations of Cu-As and As-Cd in water. In addition, pH and DO were extremely significantly positively correlated (*p* < 0.01) in maricultural water.

### 3.3. Environmental Pollution Evaluation

**Figure 4:**
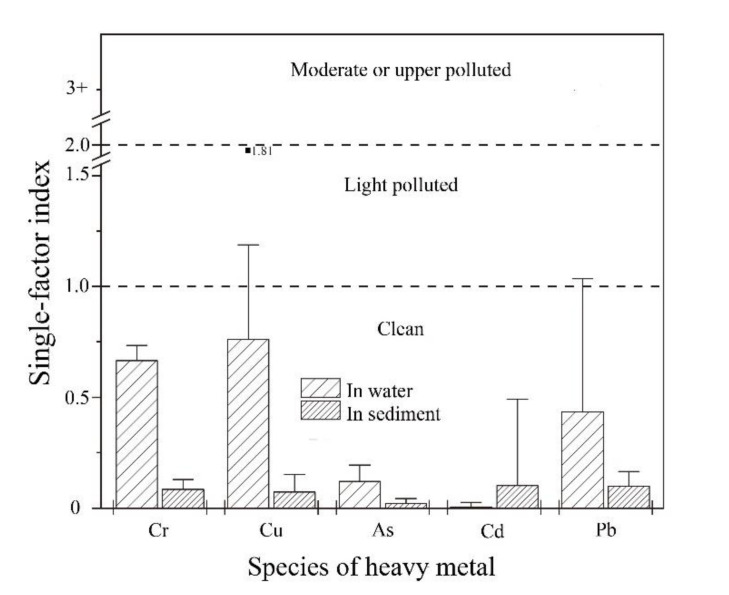
Evaluation of the single-factor index.

The SFI was used to evaluate the environmental exposure values of the five metals in water and sediment at all stations except for the sediment of the outfall station, and the results are shown in Figure 4. The mean SFIs for the metals in water decreased in the order of Cu (0.78) > Cr (0.67) > Pb (0.40) > As (0.13) > Cd (0.01), and the ranges were (0.36–1.81), (0.64–0.73), (0.07–1.35), (0.04–0.33), and (0–0.01), respectively. The Cr, As, and Cd levels all indicated no pollution in the breeding water, the Pb concentration reflected slight pollution at the outfall (O) and in pond #1 (P1), and the Cu concentration indicated slight pollution at the outfall (O) and in cage #2 (C2). The ranking of the mean SFIs for the metals in sediment was in the order of Pb (0.090) > Cr (0.084) > Cd (0.082) > Cu (0.078) > As (0.020), and the ranges were (0.044–0.136), (0.034–0.129), (0.002–0.353), (0.030–0.152), and (0.009–0.039), respectively, with all concentration values indicating no pollution.

The ecological risk posed by all metals in the sediments was low, but that of Cu in water was remarkable. On the basis of the SFI, the impact of Cu was not prominent, specifically, there was only pollution at a few stations, because the limit of Cu was based on the background level of Cu in the South China Sea (0.05 μg∙mL^−1^). If the sea water quality Class II standard (GB3097-1997) for fisheries (0.01 μg∙mL^−1^) was chosen as the limit, different degrees of Cu pollution would have been found at all stations and the proportion of seriously polluted sites would be as high as 44%.

### 3.4. Ecological Risk Assessment

All data about the chronic toxicity caused by heavy metal in marine organisms were derived from the ECOTOX Knowledgebase of USEPA and were used to evaluate the HQ. The no-observed-effect concentration (NOEC) was used as the main observation endpoint, and the maximum acceptable toxicity concentration (MATC) and lowest observed effect concentration (LOEC) were used as supplements. The geometric mean was used when multiple parallel data were available for a species [41]. The test conditions were the laboratory, salt water, and chronic toxicity. The toxicity data met the three trophic levels and five or more species (China) and also satisfied the ‘three-phylum and eight-family’ criteria of the USEPA (1985). According to the principles of reliability, pertinence and appropriateness [42], 355 toxicity data points were acquired, with 137, 59, 52, 39, and 68 data points for Cu, Cr, Pb, As, and Cd, respectively. The chronic toxicity data of the metals and the environmental exposure data of the metals fit a log-logistic distribution (*p* > 0.05). The HC_5_, PNEC, HQ_M_, and maximum hazard quotient (HQ_max_) values of the metals are shown in Table 2.

The HQ_max_ of As was less than 1, while the HQ_M_ values of the other metals were greater than 1. As a result, the ecological risk posed by As was acceptable at all stations, while the other four heavy metals posing unacceptable ecological risks. The HQs of the metals were decreased in the order of Cu > Cr > Pb > Cd > As.

**Figure 5:**
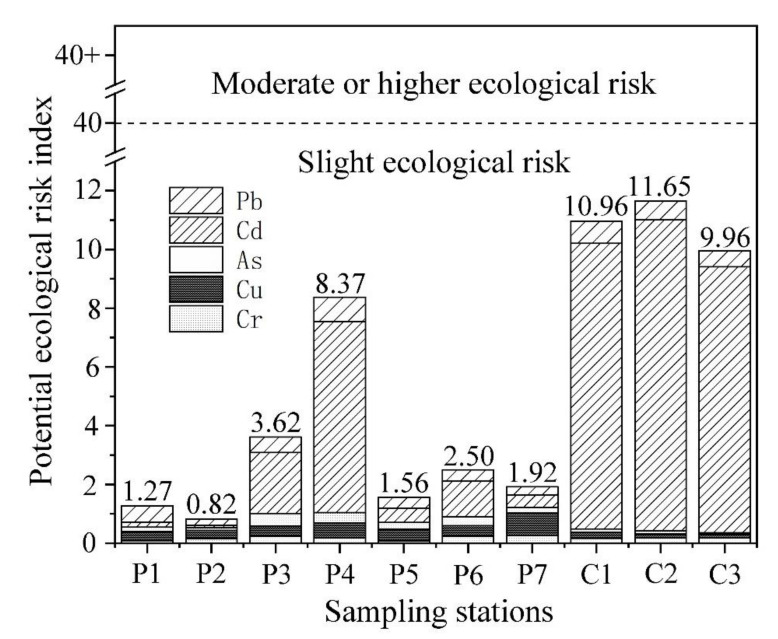
The potential ecological risk for five metals in sediment.

The analysis of the potential ecological risk index for the metals in the maricultural area is shown in Figure 5. The sediment at all stations in the maricultural area had low ecological risk. The ecological risks of the mean concentrations of the five metals were Cd (4.0) > Pb (0.51) > Cu (0.35) > As (0.20) > Cr (0.17). The potential ecological risk index of the sediment samples in seven farming ponds and three breeding cages in Jida Harbor were ranked in the order of C2 (11.65)> C1 (10.96) > C3 (9.96) > P4 (8.37) > P3 (3.62) > P6 (2.50) > P7 (1.92) > P5 (1.56) > P1 (1.27) > P2 (0.82). The potential ecological risk index in the sediment from the cage breeding area was significantly greater than that from the breeding pond area (*p* < 0.01), and all areas had slight ecological risks.

**Figure 6:**
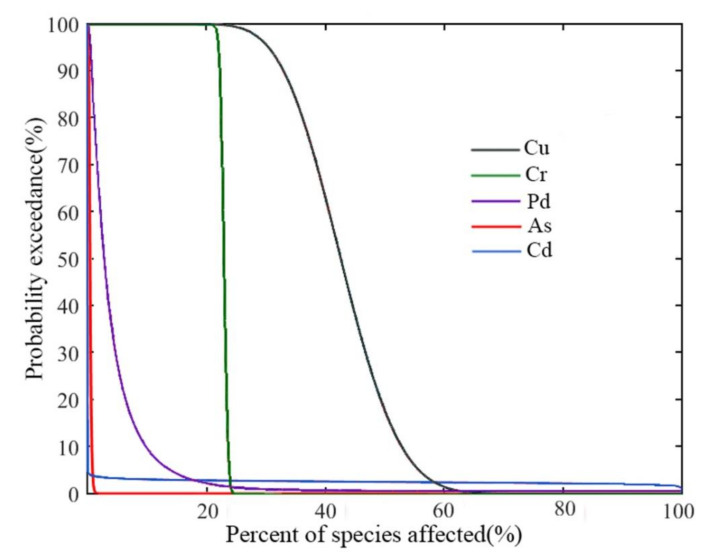
JPCs of the five heavy metals.

The ORPs of the metals from the evaluation of the JPCs followed the sequence of Cu (0.3234) > Cr (0.2277) > Pb (0.0431) > Cd (0.0253) > As (0.0050). The ORPs of Pb, Cd, and As were below the general protection level of 0.05, indicating that the ecological risk posed by Pb, Cd, and As was acceptable; the ORPs of Cu and Cr were greater than 0.05, indicating that the ecological risk posed by Cu and Cr was unacceptable (Figure 6). The HQ_max_ and ORP of Cu reached 118.73 and 0.3234, respectively, and the ecological risk posed by Cu was 1~2 orders of magnitude higher than that posed by the other heavy metals. This is likely a consequence of the heavy use of copper disinfectant and feeds with high Cu in the maricultural area, but the high background value of Cu is a factor that cannot be ignored. Therefore, Cu content limits should be added to the production standards for fish feed, and disinfectants with low Cu concentrations should be popularized and used; moreover, clay minerals and chlorella should be added to breeding water to properly reduce the heavy metal concentrations in local water areas. If horizons are sufficiently expanded, research in other fields may provide new ideas for the control of heavy metal pollution in seawater. In the presence of the complexing agent EDTA from aqueous solutions, the application of commercially available ion exchangers is one possible approach for the removal of the heavy metals Pb and Cd [43]. In addition, Pyrolox™, which contains manganese nano oxides, is used to remove Cu, Cd, Pb, and so on [44]. The new nanomaterials are promising for treating heavy metal pollution in local water areas.

The pollutant toxicity data were distributed in a relatively small interval of the JPCs. Assuming that the toxicity data were both representative and scientific, a small decreasing interval reflects a concentrated toxicity threshold of a metal to different species. Therefore, it is possible to assess whether the particular levels of metals are suitable for specific breeding activities in a specific sea area. This principle can also be applied to identify local sensitive species that are sensitive and urgently need protection and for realizing immediate ecological risk warnings and legal protection. However, all ecological risk assessment methods based on probabilistic algorithms have certain limitations. For instance, marine environmental factors (such as temperature, salinity, suspended solids) and other land-sourced pollutants (microplastics, acidic wastewater) greatly impact the form and migration of metals and ultimately impact the biological effectiveness and toxicity to marine organisms [45]. In addition, hydrography, monsoons, circulation, and dynamics are key factors that influence metal speciation and migration [17,46,47]; for example, the northwest monsoon prevails in October in China, and extremely small vortex regions easily form around the channel and prevent pollutants from dispersing. The sensitivity of different species to the same chemical varies and setting environmental protection thresholds based on toxicity data from autochthonous species is a more reliable approach for ecological protection. Appropriate breeding subjects must be selected according to the breeding environment, and environmental impacts on organisms should be excluded as much as possible.

## 4. Conclusions

In 2018, 33 water samples, 30 sediment samples, and nine feed samples were collected from a typical mariculture area in western Guangdong, and the concentrations and distribution characteristics of Cr, Cu, As, Cd, and Pb in all the samples were analyzed. The SFI, potential ecological risk index, HQ, and JPC were used to comprehensively analyze and study ecological risk in the breeding area in depth. Several certain conclusions were drawn:

(1) The concentration ranges of Cu and Pb in seawater and sediment were all wide, but those of the As and Cd all were narrower than those of the other metals. The concentration ranges of Cu were 454 times wider than that of Cd in water, and the concentration ranges of Pb were 35 times wider than that of Cd in sediment. In the feed, the Cu content was generally high and varied, but the concentration ranges of the other metals were narrow. The concentration range of Cu was 19.33–107.31 μg∙g^−1^. The contents of Cu and Pb in the breeding wastewater exceeded the standard limits at several stations.
(2) The results of Pearson correlation analysis showed that Cr, Cu, and As had similar sources (*p* < 0.01). The levels of all the metals were positively correlated in the water to a certain extent. The metal contents were heavily impacted by pH and DO in the water (*p* < 0.01), possibly causing significant differences in the contents of Cu in the farming ponds and breeding cages. Pb and Cd had the same chemical deposition properties or analogous biogeochemical behaviors in sediment (*p* < 0.05). Moreover, Cd, Cu, and As levels were extremely significantly positively correlated in feed (*p* < 0.01), probably owing to the addition of feed additives, which may contribute to the significant correlations of Cu-As and As-Cd in breeding water.
(3) The SFI and the potential ecological risk index revealed that the sediment samples collected from the studied areas all had no metal pollution and low ecological risk. At very few stations, the metal concentrations of metals Pb and Cu were indicative of slightly polluted in the water. The evaluation results of hazard quotient and joint probability curve showed that the ranking of ORPs of the metals was in the order of Cu (0.3234) > Cr (0.2277) > Pb (0.0431) > Cd (0.0253) > As (0.0050), and that for mean HQs was in the order of Cu (67.96) > Cr (41.75) > Pb (4.76) > Cd (2.21) > As (0.26). The ecological risks of Cu and Cr were unacceptable based on the JPCs and HQs. Although the HQ_M_ of Pb and Cd were greater than 1, the levels of these metals were still acceptable because their ORPs were all less than 0.05. As a major pollutant, metal copper is worth continuing to monitor and further evaluate.

## Figures and Tables

**Table 1:** The relationships among $E_{j}^{i}$, RI*_j_*, and their ecological risk levels.

$E_{j}^{i}\;\mathrm{and}\;\mathrm{Level}$		RI*_j_* and Level	
$E_{j}^{i}$ < 40	Slight	RI*_j_* < 150	Slight
40 ≤ $E_{j}^{i}$ < 80	Moderate	150 ≤ RI*_j_* < 300	Moderate
80 ≤ $E_{j}^{i}$ < 160	Strong	300 ≤ RI*_j_* < 600	Strong
$E_{j}^{i}$ ≥ 160	Serious or higher	RI*_j_* ≥ 600	Serious

**Table 2:** Evaluation of the HQs of the metals.

Metal Type	Cu	Cr	Pb	As	Cd
HC_5_(μg∙mL^−1^)	0.0025	0.0079	0.0204	0.0617	2.37 × 10^−4^
PNEC(μg∙mL^−1^)	0.0005	0.0016	0.0041	0.0123	4.74 × 10^−5^
HQ_M_	67.96	41.75	4.76	0.26	2.21
HQ_max_	118.73	44.22	22.43	0.47	2.78

## Data Availability

The data presented in this study are available on reasonable request from the corresponding author. The data are not publicly available due to privacy or ethical considerations.
